# Occurrence of *Trichinella* spp. infestations in black bears in Quebec, Canada

**DOI:** 10.1177/10406387261422686

**Published:** 2026-04-09

**Authors:** Simon Krückemeier, Christopher Fernandez-Prada, Marie-Odile Benoit-Biancamano

**Affiliations:** Ecology and Emergence of Zoonotic Diseases, Helmholtz Institute for One Health, Greifswald, Germany; Groupe de recherche sur les maladies infectieuses en production animale, Centre de diagnostic vétérinaire de l’Université de Montréal, Département de pathologie et microbiologie, Faculté de médecine vétérinaire, Université de Montréal, St-Hyacinthe, Québec, Canada; Groupe de recherche sur les maladies infectieuses en production animale, Centre de diagnostic vétérinaire de l’Université de Montréal, Département de pathologie et microbiologie, Faculté de médecine vétérinaire, Université de Montréal, St-Hyacinthe, Québec, Canada

**Keywords:** game meat, nematodes, parasites, public health, *Trichinella*, *Ursidae*, zoonoses

## Abstract

*Trichinella* spp. are parasitic nematodes with a worldwide distribution that infect mammals, birds, and reptiles. *Trichinella* is composed of at least 13 taxa; the latest taxon, T13, was identified in wolverines in northwestern Canada in 2020. *Trichinella* is of public health importance because trichinellosis (trichinosis) is a zoonosis transmitted by eating undercooked meat from wild or domestic animals. In Canada, wild game species are the main source of infection; outbreaks in people originate almost exclusively from consumption of bear and walrus meat. Black bears (*Ursus americanus*), with a population of ~70,000 individuals, are widespread in Quebec; they are a commonly hunted species and a potential source of infection. To estimate the incidence of this parasite in the black bear population, we collected 250 tongue samples from 14 different Quebec regions. A single sample from a female black bear from the Côte-Nord region was positive, resulting in an estimated prevalence of 0.4% (95% CI [0.01, 2.21]). The larval content of >5 larvae/g in this sample was 5 times the minimal infectious dose for humans. Hence, although the prevalence of *Trichinella* spp. in our survey of black bears in Quebec was relatively low, clinical manifestation in humans is likely to occur when eating meat from such an infected animal, underscoring the need to follow the recommended cooking practices before consuming bear meat.

*Trichinella*, a parasitic nematode in the *Enoplea* class,^40^ is found around the globe, with the exception of Antarctica, which has not reported any occurrence of this parasite.^38^ Long considered to consist of only 1 species, this genus is now known to be composed of at least 13 different taxa, identified at the species or genotype level.^32^ The last taxon, *Trichinella chanchalensis* (T13), was identified in wolverines in northwestern Canada in 2020.^47^ *Trichinella* spp. are divided into 2 clades, depending on whether a capsule is formed in the infected muscle cells or not. Among the various clades, the encapsulated group stands out as the largest, encompassing 7 distinct species: *T. britovi*, *T. chanchalensis*, *T. murrelli*, *T. nativa*, *T. nelsoni*, *T. patagoniensis*, and *T. spiralis*; and 3 genotypes: *Trichinella* T6, *Trichinella* T8, and *Trichinella* T9—all of which exclusively infect mammals.^32,40^ The formation of a capsule (composed of a nurse cell and a collagen wall) around a larva is a complex process.^51^ The nonencapsulated clade includes *T. pseudospiralis*, infecting mammals and birds, and *T. papuae* and *T. zimbabwensis*, infecting mammals and reptiles. Canada is home to 6 of these species and genotypes: *T. murrelli*, *T. nativa*, *T. pseudospiralis*, and *T. spiralis*, as well as *Trichinella* T6 and *T. chanchalensis*.^40,47^

Natural hosts vary among the different taxa of *Trichinella*, with differences in the epidemiology of the infection among host species.^37^ Thus, bears are not susceptible to every *Trichinella* species and genotype, and differences also exist among the various bear species. Based on molecular or biochemical identification, grizzly bears are considered natural hosts for *T. spiralis* (T1), *T. nativa* (T2), and *Trichinella* T6; polar bears are hosts for *T. nativa*, *T. pseudospiralis* (T4), and *T. spiralis*; and black bears are hosts for all 4 of the above-mentioned taxa (T1, T2, T6, T4).^39^ In other studies, *Trichinella* T6 and *T. nativa* were reported most commonly.^17,27,48^

*Trichinella* is the etiologic agent of trichinellosis (trichinosis), a zoonotic disease transmitted through the consumption of inadequately cooked meat sourced from various infected domestic or wild carnivores and omnivores, as well as certain herbivores. *T. nativa* and T6 have a particular clinical significance in the northern regions (*T. nativa* was found in the coldest regions of the Holarctic; T6 has only been detected in North America),^40^ as they are resistant to freezing—increasing their zoonotic risk because conventional freezers do not efficiently kill the encysted larvae.^31^ They are also the most common taxa in the Nearctic region.^49^ Consequently, both taxa are the predominant species in the subarctic and arctic regions of Canada, and *Trichinella* T6 is the most observed genotype in Canadian wildlife, with a wider geographic distribution and host range than *T. nativa.*^17,48,49^ Trichinellosis is recognized as one of the most significant foodborne diseases, with documented human infections and outbreaks spanning at least 55 countries.^39,43^ The risk of trichinellosis is moderated by eating habits and food preferences, as well as by religious and cultural practices, which also can influence the consumption of meat from various wildlife species.^43^ Hunting practices and traditional methods of food preparation still play a central role in human exposure and the potential spread of *Trichinella* in the northern regions of North America.^24^

Two distinct *Trichinella* cycles, the domestic and the sylvatic cycles, are described. The domestic cycle mainly includes pigs, horses, and rodents; the sylvatic cycle includes several wildlife or feral mammals, reptiles, and birds.^6^ Domestic and wild animals, especially carnivores, act as reservoirs. Epidemiologically, domestic and sylvatic carnivores are essential, as almost all *Trichinella* species can complete their life cycle in these animals.^37^ Infectivity and survival of *Trichinella* larvae in muscle tissue vary from a few months to several years depending on the host and its environment (lifespan, size, position in the food chain, etc.).^39^ For instance, in polar bears, which are considered highly adequate reservoir hosts, *T. nativa* larvae were found to be infective for up to 20 y and to survive for at least 5 y in muscle frozen at −18°C.^25,40^

Trichinellosis is not well documented in animals, and clinical manifestations seem to be rare.^18,42^ In humans, trichinellosis is a serious and sometimes fatal disease.^9^ Clinical manifestations increase in severity according to the parasite burden; although the minimum infectious dose of larvae has not been clearly defined, estimates have been made.^11^ Empirically, the ingestion of meat containing at least 1 larva/g (lpg) was determined as a threshold for clinical manifestation, corresponding to an infective dose of ~150 larvae for a normal meat consumer (presuming an average meat consumption of 150 g).^11^ Additionally, it is believed that the infection becomes clinically relevant with ~10 lpg of human muscle biopsy, and is severe with >100 lpg. Therefore, an intake of 100–300 larvae can be estimated as the minimal infective dose.^11^ Other sources consider ingestion of 70 larvae sufficient to cause clinical disease.^11^

The human course of infection is divided into 2 phases: an intestinal (or enteral) phase and a muscle (or parenteral) phase.^18,43^ In the intestinal phase, larvae are released through the digestion of the ingested muscle and the larval capsule in the stomach,^50^ which allows larvae to penetrate the mucosa of the small intestine, reach adult age within 48 h post-infection, and to mate.^18^ Female worms then release larvae in lymphatic and blood vessels from 5 d post-infection onward (larval production can last from 1 to several weeks, depending on the host’s immunity).^18^ In the muscle phase, the new larvae reach and penetrate striated muscle cells. They transform the muscle cell into a nurse cell (which provides nutrition and protection and in which they can survive for years) and eventually grow to become “mature” infective larvae.^18,50^ Encapsulated *Trichinella* spp. induce a collagenous capsule and after a period of time (weeks, months, or even years), mineralization may occur.^18^ Nonencapsulated species, such as *T. pseudospiralis*, do not encapsulate after invading the muscle cell and instead remain free in the nurse cell.^50^ Abdominal pain and diarrhea occur in the intestinal phase; fever, periorbital edema, and myalgia can manifest in the muscle phase, with possible complications, such as myocarditis, thromboembolic disease, and encephalitis.^13^ Early and late clinical diagnosis of the disease is difficult—pathognomonic symptoms and signs are lacking in the early stage and physicians in nonendemic countries may not have experience with the pathogen.^18^

Historically, the zoonotic risk was mostly attributed to *T. spiralis* through domestic pig farming,^52^ but modernization of production systems, strict surveillance programs in slaughterhouses, as well as the decrease of free-range and backyard pig farming considerably reduced domestic pork meat as a source for *Trichinella* infections in humans.^43^ In Canada and the United States, *Trichinella* is considered eradicated from commercial confinement-raised pigs entering the food chain.^18,39^ In Canada, the last report of trichinellosis originating from pigs occurred in Ontario in 2013 from animals on a non-commercial farm.^35^ With the decrease of pig meat as the origin of human trichinellosis, the importance of infected game meat has increased in many countries; wild boar and bear, as well as other carnivore species, play a notable role.^6,43^ In Canada, documented outbreaks are almost exclusively linked to bear and walrus meat,^27^ with an annual incidence of 10–15 cases.^39^ A study estimated the seroprevalence for *Trichinella* at 18.6% in the Canadian Inuit population.^21^ Occasional hunters and travelers visiting regions where bear meat is consumed are also at risk.^12^

Bear meat in North America is used mainly for human consumption.^43^ In recent years, bear hunting has grown in popularity. In Quebec, where an estimated 70,000–75,000 black bears (*Ursus americanus*) live,^20^ the government recorded >18,000 licenses sold in 2018 and >6,500 animals harvested by hunting or trapping, reaching record levels (Ministère de l’Environnement, de la Lutte contre les changements climatiques, de la Faune et des Parcs, pers. comm., 2023 Jul 05).^30,33^ In 2022, the number of hunted and trapped black bears was >5,000.^19^

Trichinellosis is the most commonly reported zoonosis from bears, with the first reports dating from the 1930s.^8^ Thus, determining the prevalence of *Trichinella* spp. in bears from different geographic regions is essential to assess the zoonotic risk linked to meat consumption. Past outbreaks of trichinellosis after meat consumption from black bears originating from Quebec strengthen the seriousness of the zoonotic risk, the need to take adequate precautions, and to conduct research in this regard.^1,26^ In the Nearctic region, black bears and red foxes are the 2 most extensively researched host species of *Trichinella*.^6^ Over the past several decades, different studies have quantified the prevalence of *Trichinella* spp. in Canadian wildlife, documented human trichinellosis outbreaks, and established national surveillance programs to monitor the parasite.^7,22,27,28,45^ Only a few studies have estimated the prevalence of *Trichinella* spp. in bears in Quebec. A 1977 report found that 1 of 107 black bear diaphragms examined were positive.^15^ A 2006 project estimated a prevalence of 0.9% in black bears in the province.^5^ Beyond the 1977 and 2006 Québec-focused studies, only one national survey incorporated black bears from the province in 2010. That investigation analyzed carnivore muscle samples that were collected across Canada for more than a decade to estimate overall *Trichinella* prevalence and identify circulating species and/or genotypes.^17^ Larvae were found in 14 of 193 black bears (7.3%), but the authors did not indicate how many of those animals came from Québec, nor did they report region-specific prevalence or larval burdens.

Our objective was to determine the occurrence of *Trichinella* spp. infestation in black bears in Quebec and to identify the regions with the highest infestation levels.

## Materials and methods

In 2018, 250 tongue samples from black bears were provided to the Veterinary Diagnostic Center of the Université de Montréal (CDVUM; St-Hyacinthe, Québec, Canada). The chosen sample size was based on a 2006 study that also assessed the prevalence of *Trichinella* in black bears in Quebec.^5^ Most samples consisted of the whole tongue (in a few cases, the tongue was incomplete), and all descriptive data submitted by the contributor were collected (i.e., hunting site, animal size, sex). Tongues were kept frozen at −18°C before downstream analysis.

For each pooled sample, equal masses of tongue muscle were excised from 3 bears. When available, 20 g was collected from each tongue (total 60 g). The detection method was based on a previously described peptidic digestion, which has been approved by the American Association of Veterinary Laboratory Diagnosticians.^14^ If <20 g was obtained from 1 tongue, the amount taken from the other 2 tongues was reduced to the same weight. Pools testing positive were subsequently deconvoluted by digesting each of the 3 tongues individually to identify the infected animal. Briefly, to recover L1 larvae from the samples, 60 g of tongue muscle was minced into thin strips, blended with pepsin-HCl (0.5% w/v or 1% v/v; Fisher), and transferred to a beaker. The mixture was agitated at 45°C for 90 min, then passed through a 180-μm sieve. The filtrate was allowed to settle for 30 min, after which 125 mL of sediment was collected; the decantation step was performed a second time to maximize larval recovery. A 25-mL aliquot of the settled digest was transferred to a Petri dish and examined under a stereomicroscope. All L1 larvae were enumerated, and the larval burden (expressed as lpg) was calculated by dividing the total larval count by the weight of tissue digested.

The prevalence of *Trichinella* spp. was calculated with Wilson score 95% CIs. All analyses were carried out with R (https://www.R-project.org). The total number of hunted black bears in 2018 was obtained through personal communications from the Ministère de l’Environnement, de la Lutte contre les changements climatiques, de la Faune et des Parcs (MELCCFP–Ministry of Environment, Fight Against Climate Change, Wildlife and Parks), Gouvernement du Québec, and corresponded to those published in several newspapers.^29,30^

## Results

**Figure 1.**
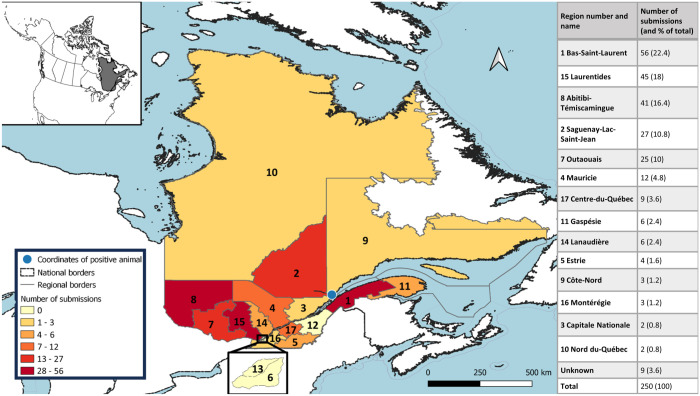
Map of Quebec, Canada, with the number of sample submissions per region. The positive female black bear was hunted in region 9, Côte-Nord. The table on the right indicates the number and the percentage of submissions per region out of the total number of submissions. Map created with QGIS 3.28.10 Firenze (https://qgis.org/).

The province of Quebec is subdivided into 29 regions; we received samples from 14 regions (**Fig. 1**). More samples were from males (113 of 250; 45.2%) than from females (49 of 250; 19.6%), although the sex of the animal was unknown or not specified for 88 samples (35.2%).

**Figure 2.**
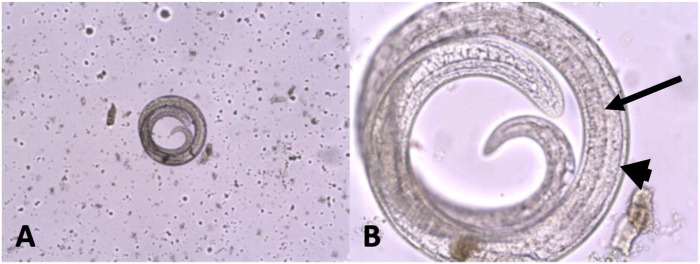
*Trichinella* parasite from the positive female black bear. **A.** Coiled *Trichinella* sp. larvae are easily recognized at 100×. **B.** The muscular wall (arrowhead) and digestive tract (arrow) are visible at higher magnification (original objective 400×).

Of the 250 submitted samples, only 1 was positive (0.4%; 95% CI [0.02, 2.23]; **Fig. 2**, **Table 1**). The specimen was from an adult female black bear in the Côte-Nord region. The sample contained >5 lpg of muscle tissue. In the 2006 study, *T. nativa* was the species found in all animals. Thus, *T. nativa* could be the suspected taxon infecting the positive black bear in our study. However, DNA extraction and subsequent sequencing were not carried out to confirm this hypothesis.

**Table 1.** Nominative and quantitative information from 2 studies on the occurrence of *Trichinella* spp. in black bears in Quebec.

	2006 study^5^	Our 2018 study
Total bears killed that year	4,788	6,513
Sample size (%)	659 (14)	250 (3.8)
No. of positive cases	6	1
Prevalence	0.9%	0.4%
Region of origin of positive cases	Côte-Nord; Abitibi-Témiscamingue; Lanaudière; Laurentides; Mauricie; Outaouais	Côte-Nord
Larval burden in muscle (larvae/g)	0.01–4.15	>5
Samples >20 g	9%	22.8%
% males/females/unknown	58/33/9	45/20/35

## Discussion

In our survey of 250 black-bear tongues harvested in 14 of Québec’s 29 administrative regions, we detected *Trichinella* spp. in only 1 animal, an adult female from Côte-Nord harboring >5 lpg of muscle, yielding an overall prevalence of 0.4% (95% CI [0.02, 2.23]). This point estimate is slightly lower than the 0.9% prevalence reported in the 2006 study of 340 bears^5^ and the identical 0.9% prevalence documented in the 1977 survey of 107 bears.^15^ Within most Canadian regions, the prevalence is generally <1.5%.^2^ However, the Canadian national prevalence estimated in 2 different studies was higher; 1.9% (*n* = 1,575)^2^ and 7.3% (*n* = 193).^17^ Noticeable regional differences exist; higher occurrences of this parasite have been observed in other provinces and territories. In British Columbia, a research project determined a prevalence of 12% (*n* = 192).^46^ In the Northwest Territories, researchers found a prevalence of 5.8% (*n* = 120),^27^ and, in another study, a prevalence of 4.1% (*n* = 197).^28^ A 2021 report recorded a 20% prevalence in the Yukon, the highest in Canada^22^; however, the number of samples (*n* = 30) was relatively small. Also, in the United States, the prevalence of *Trichinella* infection varies among states. The highest prevalence in North America was reported in Alaska—once in a serologic study (27.5%; *n* = 40)^4^ and once through tissue sampling (21.7%; *n* = 23).^41^ Earlier studies found that 13% of the tested animals were seropositive for *Trichinella* in Idaho (*n* = 122)^3^ and California (*n* = 141)^44^; in the neighboring state of Oregon, no animal was found positive with muscle digestion (*n* = 250), and a seroprevalence of only 2% was determined (*n* = 103).^34^ A seroprevalence of 3% (*n* = 181) was found in Pennsylvania.^10^

The larval load of *Trichinella* spp. in our positive case (>5 lpg) was slightly higher than in the 2006 research project, in which a range of 0.01–4.15 lpg was reported.^5^ On a Canadian national scale, reported larval burdens were 0.10–659 lpg.^17^ In the Northwest Territories, the concentration of *Trichinella* larvae was 0.04–177 lpg.^27,28^ In the study conducted in the Yukon, it was 0.09 to 1,173 lpg, with a mean of 401 lpg.^22^ A range of 0.5–400 lpg was found in Alaska.^41^ A larval concentration of ≥1 lpg is empirically assumed to be necessary to cause infection and induce clinical signs in humans.^11^ The positive animal in our study had 5 times the minimal infectious dose and, therefore, would be of concern for human consumption.

Our observed low prevalence of *Trichinella* among black bears in Quebec, which is significantly lower than in other provinces, could be the result of various factors, such as the geographic spread of *Trichinella* spp. and the varying infectivity and endurance of distinct strains.^2^ The parasite might be rare in Quebec, or the encountered strains might have a lower infectivity for black bears than in other regions. As sources of *Trichinella* infections are food-related, another reason could be the different diet of the animals or the low occurrence of the parasite in other carnivores and omnivores in Quebec.^17,39^ *Trichinella* is believed to be maintained in the food chain via cannibalism, predation, and especially the eating of carrion.^39^ However some black bear populations have been found to mainly forage on nuts and plants, with animal protein sources comprising only a minor part of their overall diet.^23^ Furthermore, although wild carnivores and omnivores are the hosts for *Trichinella* spp. in the sylvatic cycle and carnivores at the top of the food chain are considered the main reservoirs,^39^ the prevalence varies among species. Thus, in northern Canada, the prevalence in black bears was considerably lower than in other species, such as wolverines, grizzly bears, polar bears, and wolves.^36^ Considering the methods used in our study, a falsely low prevalence attributable to inaccurate muscle samples is very unlikely, as the tongue, (together with diaphragm and masseter muscles) is considered a predilection site for *Trichinella* larvae in bears.^16^ Furthermore, tongue muscles were the most frequently used samples in other studies.^17,22,28^

Our sample submissions were only a small percentage (3.8%) of the overall number of bears harvested in Quebec. The number of submissions varied considerably between regions, with possible over- or underrepresentation of populations in specific regions and an absence of samples from as many as half of the regions. Indeed, overall, the number of submissions was correlated with the number of black bears harvested (through hunting or trapping) in these different areas, as well as with the distribution of the black bear population in Quebec (pers. comm. from the MELCCFP, 2023 Jul 05).^33^ Thus, more tongues were sent to us from regions with higher hunting activity than from regions with lower hunting activity. Even if spread throughout the province, harvests tend to be more concentrated in the south and the center of Quebec, where population densities of black bears are also higher (especially in the central part of Quebec, with 2–3.7 animals/10 km^2^). In contrast, fewer submissions came from more northerly areas, as well as Montérégie, where the hunting and trapping activity, together with the black bear population, are lower (0–1 animals/10 km^2^). However, some regions, such as Capitale Nationale and Estrie, were surprisingly underrepresented considering the intensity of black bear harvesting in these regions. The population density of black bears in Estrie is lower (1–2 animals/10 km^2^) but is high in Capitale Nationale (2–3 animals/10 km^2^). Submissions were higher in areas with greater publicity about our project. Several officials of the *Pourvoiries* (Outfitters) or the Ministère des Forêts, de la Faune et des Parcs (Ministry of Forests, Wildlife and Parks) in those areas had even taken on the task of sending grouped samples. The low submissions in some regions with comparatively high black bear harvesting is likely related to a lack of awareness of the project.
